# Using magneto-electroluminescence as a fingerprint to identify the spin polarization and spin–orbit coupling of magnetic nanoparticle doped polymer light emitting diodes†

**DOI:** 10.1039/c9ra01501a

**Published:** 2019-05-21

**Authors:** Weiyao Jia, Tadaaki Ikoma, Lixiang Chen, Hongqiang Zhu, Xiantong Tang, Fenlan Qu, Zuhong Xiong

**Affiliations:** School of Physical Science and Technology, Southwest University Chongqing 400715 People's Republic of China zhxiong@swu.edu.cn; MOE Key Laboratory on Luminescence and Real-Time Analysis, Southwest University Chongqing 400715 People's Republic of China; Graduate School of Science and Technology, Niigata University 2-8050 Ikarashi, Nishi-ku Niigata 950-2181 Japan

## Abstract

The spin polarization and spin–orbit coupling (SOC) in polymer light emitting diodes (PLEDs) with the active layer doped with Fe_3_O_4_ nanoparticles (NPs) were identified through magneto-electroluminescence (MEL). By comparing the MEL characteristics such as linewidth and magnitude between PLEDs with and without Fe_3_O_4_ dopant, we confirmed the existence of spin polarization, but ruled out the existence of SOC. Although the spin polarization is positive to electroluminescence, the brightness–current characteristics suggested that the current efficiency of the doped PLED does not improve. We attributed it to the current leakage caused by the Fe_3_O_4_ NPs in the active layer. This work is beneficial for us to further understand the effect of magnetic nanoparticle doping on the dynamic behavior of excitons and polaron pairs in organic semiconductor devices.

## Abstract 2

The existence of spin polarization was confirmed while spin–orbit coupling was ruled out in Fe_3_O_4_-based PLEDs by magneto-electroluminescence analysis.

## Graphical abstract

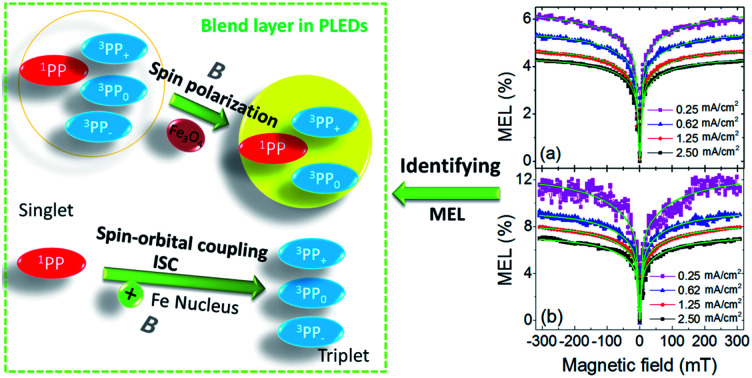

## Introduction

Ferrimagnetic nanomaterials are promising materials due to their extensive application potential.^1–6^ In recent years, they have been used in organic electronic devices, like polymer light emitting diodes (PLEDs) and organic solar cells, as anode buffers to improve the injection of holes^7–11^ or as an active layer dopant to improve the internal quantum efficiency by adjusting the number ratio of single excitons to triplet excitons (*R*_ST_).^12–15^ Two distinct views have been proposed on the direction of change in the *R*_ST_. Bin Hu and Sun *et al.* suggested that the spin polarization of metal magnetic nanomaterials promotes the conversion from triplet polaron pair (^3^PP, the precursor of the triplet exciton) to singlet polaron pair (^1^PP, a precursor of singlet exciton) in PLEDs.^14,15^ According to the spin polarization model the holes are injected from the anode into the magnetic nanomaterial dispersed in the active layer under a bias voltage, and the holes are spin-polarized by magnetic nanomaterial before they hop onto the host molecule. Different to holes, almost all the electrons are injected directly into the host molecule from the cathode without spin polarization due to the large electron barrier between magnetic dopants and the host. The recombination of fully spin-polarized holes and electron without spin polarization forms ^1^PP and ^3^PP with a branch ratio of 1/3. However, it creates unequal populations of 1/4 : 1/4 : 1/2 among three PP states: ^1^PP_*m*=0_, ^3^PP_*m*=0_ and ^3^PP_*m*=1_. These spin injection-induced unequal populations can be redistributed by mutual intersystem crossing of ^3^PP_*m*=1_ ↔ ^3^PP_*m*=0_ and ^1^PP_*m*=0_ ↔ ^3^PP_*m*=0_, leading to equal populations of 1/3 : 1/3 : 1/3, as shown in Scheme S1 (ESI†). Thus, the spin polarization injection induced by magnetic nanomaterial theoretically increases the *R*_ST_ from 1/3 to 1/2. On the other hand, González *et al.* proposed that the spin–orbit coupling (SOC) of metal atoms in magnetic nanoparticle increases the singlet–triplet conversion (intersystem crossing, ISC) leading to a decrease in *R*_ST_.^16^ If this is the case, we must avoid the negative impact of SOC when the spin polarization of magnetic dopants is used to improve the electroluminescence (EL) efficiency of PLEDs. Therefore, it is necessary to verify whether spin polarization and SOC can coexist in magnetic-nanomaterial doped organic semiconductor devices and their possible effects on *R*_ST_.

Multi-frequency electron paramagnetic resonance (EPR) has been applied to identify the presence of SOC.^17–19^ However, this technology often requires organic molecules to be isolated in solvent.^20^ In addition, the EPR method is susceptible to interference by hyperfine interaction, causing difficulties in identifying SOC in solid film. The EL of organic semiconductors always exhibits a response to an external magnetic field, which is known as magneto-electroluminescence (MEL). The MEL responses originated from internal spin interaction processes in organic semiconductors.^21–27^ Different spin interaction processes generally have different MEL characteristics such as line shape and linewidth. Thus, MEL can be served as “characteristic fingerprints” to identify the dynamic behaviours of underlying spin interactions in a non-destructive manner.^28–30^

Generally, the SOC-induced energy level splits (intrinsic Zeeman effect) is ∼100 μeV in molecular materials containing heavy metals.^31^ Thus the MEL response of SOC is observable only when the external magnetic field exceeds 1000 mT.^32–34^ However, the strength of SOC weakens rapidly with increasing radius of interaction or decreasing atomic number of the metal. If the atomic number of the metal is small or the metal atoms are not inside the organic molecule, the SOC strength will be greatly weakened.^32^ In this case, both the SOC and HFI are easily suppressed by a magnetic field leading to an appreciable positive MEL response.^33^ Unlike SOC and HFI, spin polarization effect is enhanced by an external magnetic field due to the magnetization of magnetic nanomaterials. This also generates a positive MEL.^14,15^ However, SOC has a larger characteristic magnetic field than HFI.^35,36^ The favorable magnetic field for the spin polarization should be different to the characteristic magnetic fields for HFI and SOC. Therefore, using MEL as a fingerprint is a simple and feasible way to simultaneously identify the spin polarization, SOC, and HFI in a solid film.

In this paper, the MEL responses were used to identify the SOC and spin polarization in PLEDs based on Super Yellow-phenylenevinylene SY-PPV/Fe_3_O_4_ blends. The SY-PPV was chosen as the active layer because it is an excellent HFI-dominant polymer host both for PLEDs and solar cells.^37–40^ The line shape and magnitude of MEL indicate that there is spin polarization in the device but no SOC. The current–brightness–voltage (*J*–*B*–*V*) characteristic curves suggest that the Fe_3_O_4_ NPs act as current-leakage centers leading to a serious reduction in current efficiency.

## Results and discussion

### EL spectrum

**Fig. 1:**
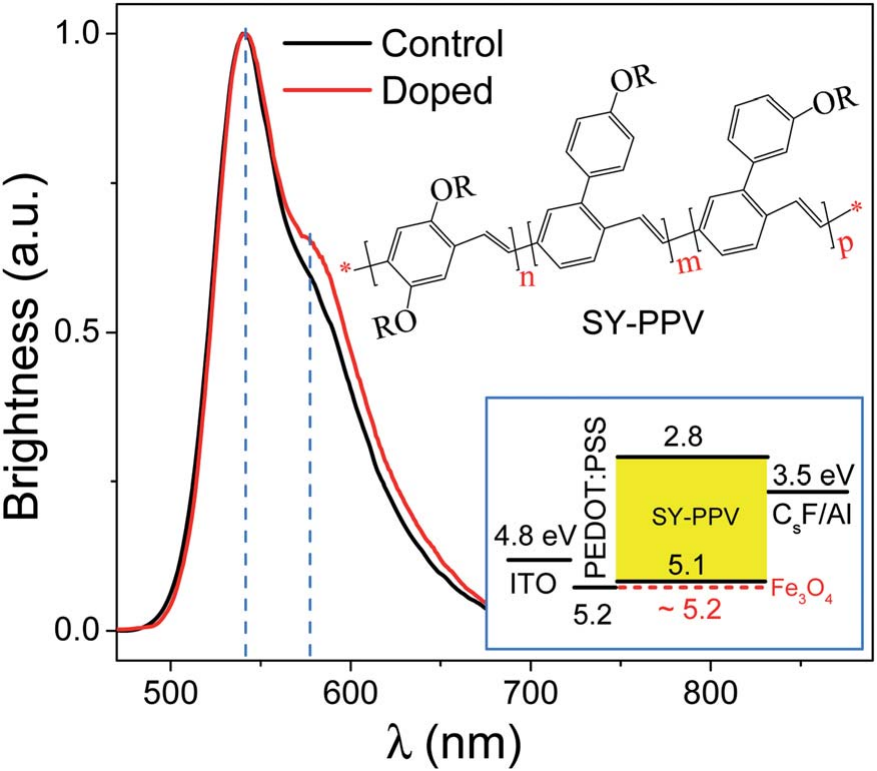
EL spectrum of pristine SY-PPV based control device (black solid line) and SY-PPV:Fe_3_O_4_ device (red solid line). Inset: SY-PPV molecular structure and diagram for the device structure and energy level arrangement of ITO, PEDOT:PSS, SY-PPV, Fe_3_O_4_ and CsF/Al electrodes.

The EL spectra of the control device and the doped device were measured respectively, as shown in Fig. 1. The insets are the molecular structure of SY-PPV and the diagram for the architecture and energy level arrangement of the doped device. The LUMO and HOMO levels of SY-PPV are −2.8 and −5.1 eV, respectively.^25^ The Fermi level of Fe_3_O_4_, ∼5.2 eV,^41^ is almost the same as the work function of PEDOT:PSS. Thus, Fe_3_O_4_ can be used as an effective electron acceptor for SY-PPV to obtain P-type doped PLEDs.^11^ The CsF/Al composite electrode with a work function of ∼3.5 eV can well reduce the electron injection barrier.^42^ Both the doped and control devices have a main peak of EL spectrum, 542 nm (2.29 eV), which is consistent with the main peak (2.30 eV) of PL spectrum of pristine SY-PPV.^43^ It should be noted that the doped device has a slight broadening EL spectrum relative to the control device from the position near the shoulder peak to the right side, but the shoulder peak (∼578 nm, ∼2.15 eV) did not red shift. This indicates that the contribution from 0–1 radiation is slightly enhanced by the doped Fe_3_O_4_ NPs which, however, did not obviously change the molecular structure of the host material. The EL still comes from the de-excitation radiation of the SY-PPV molecule.

**Fig. 2:**
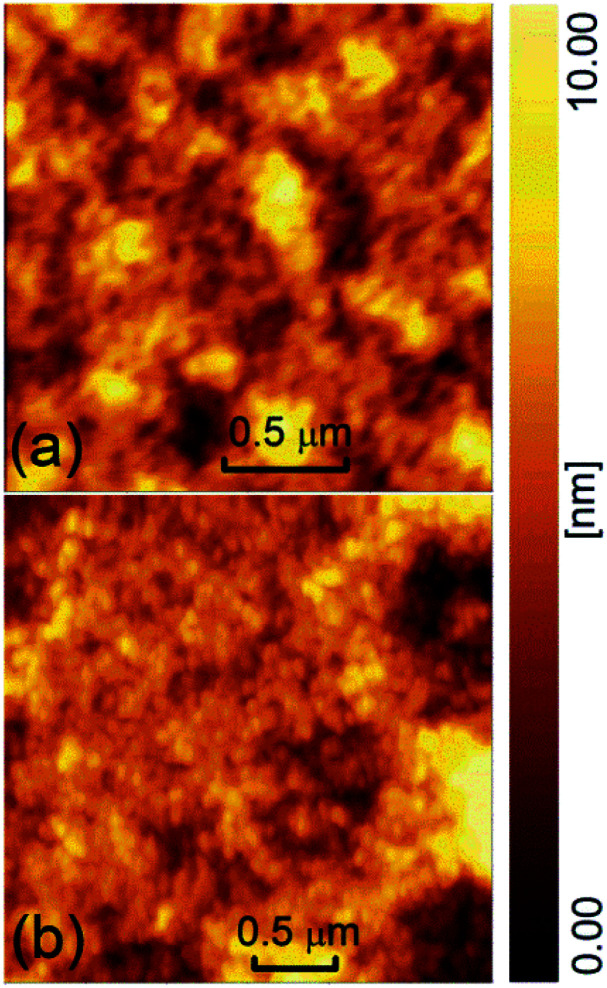
The AFM of the active layer of (a) control device and (b) doped device.

In order to evaluate the surface morphology and microstructure of the active layers, we measured their AFM both in control and doped devices, as shown in Fig. 2(a) and (b). The pale yellow is the SY-PPV molecule in the amorphous film. The dark brown areas should be the pinholes formed by the accumulation of impurities or NPs. The pristine and blend layers preserved similar morphology, the former having a Root-Mean-Square (RMS) roughness of ∼1.54 nm and the latter being ∼1.70 nm.

**Fig. 3:**
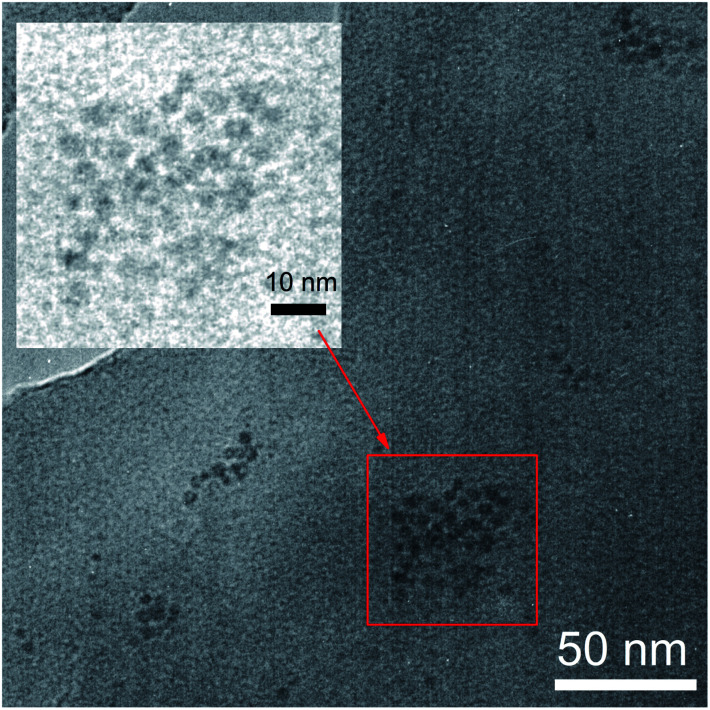
TEM image of 5 nm-Fe_3_O_4_ NPs trapped in SY-PPV. The sample of blend layer was placed on a copper TEM grid for examination.

To verify the Fe_3_O_4_ NPs trapped in the polymer, transmission electron microscopy (TEM) analysis of SY-PPV/Fe_3_O_4_ blend film was performed and the results were shown in Fig. 3. It suggested that the Fe_3_O_4_ NPs unevenly distributed in the polymer. They tend to aggregate into small separated islands 10–30 nm in size and spaced 50–100 nm apart. The Fe and O peaks in corresponding Energy Dispersive X-ray (EDX) spectra (Fig. S1†) further confirmed that the iron oxides NPs were doped successfully into the polymer.

**Fig. 4:**
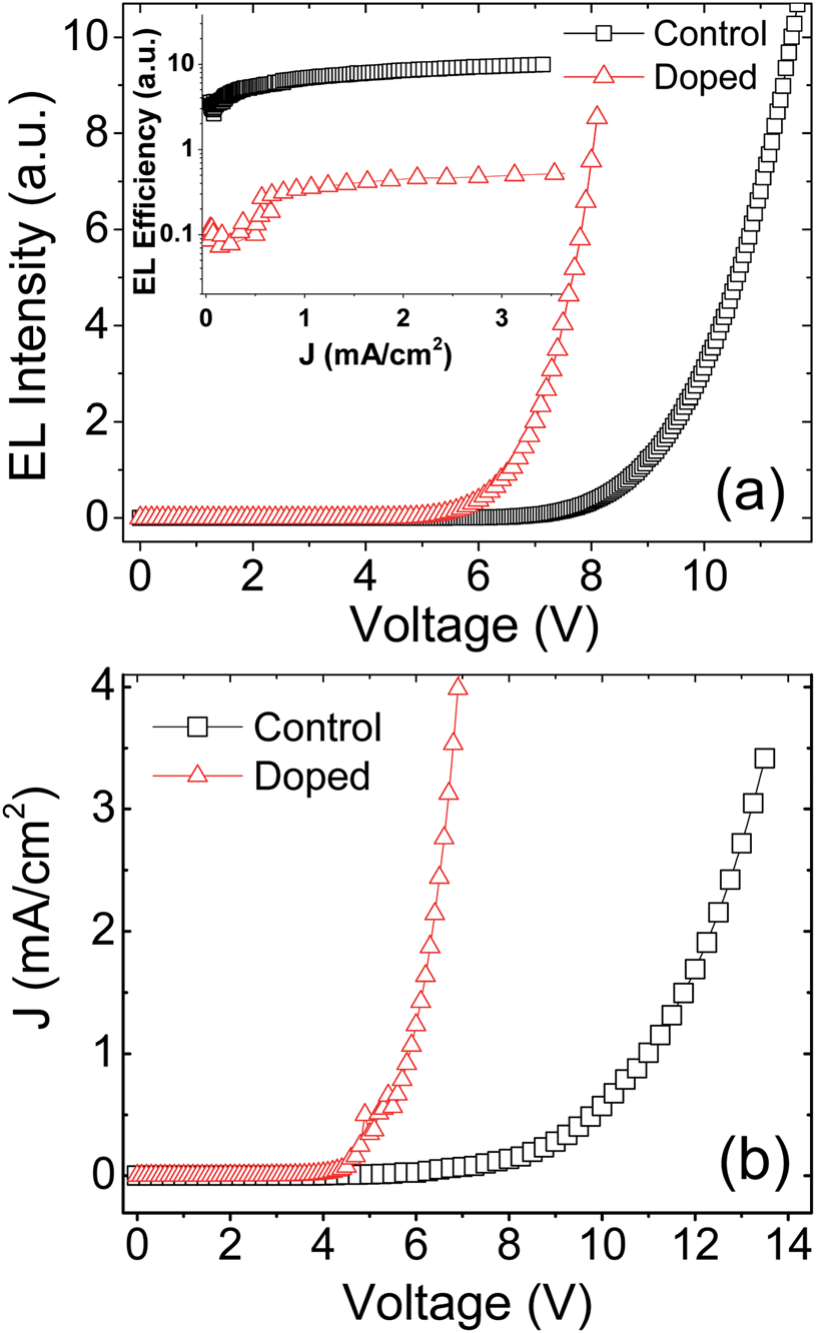
Experimental *B*–*V* (a) and *J*–*V* characteristics (b) of doped and control devices. The inset of (a) is *J* dependence of EL efficiency.

The brightness–voltage (*B*–*V*) and current–voltage (*J*–*V*) characteristic curves of control and doped devices were plot in panel (a) and (b) of Fig. 4, respectively. Panel (a) shows that the turn-on voltage of the doped device is much lower than the control device. Unexpectedly, the EL efficiency (inset) of the former is about one-twentieth of the value for the latter over the current density range. The *J*–*V* characteristics in panel (b) indicated that the doped device has a remarkable higher current density above the turn-on voltage than the control device. However the *J*–*V* curves exhibit typical semiconductor characteristics. In addition, because the centrifugal force generated by spin coating is in the plane direction of the film, the size of these aggregates should be slightly smaller in the normal direction (direction of applied electric field) than the 10–30 nm in plane direction. Thus the aggregates can hardly generate short circuit by penetrating the blend film. Thus we can safely eliminate the possibility of short-circuit currents.

In our opinion, the poor EL efficiency of the doped device is probably due to the large leakage current caused by the aggregate of Fe_3_O_4_ NPs. First, the electrode-landed aggregates tend to make the film localized thinner,^44^ forming Fowler–Nordheim (F–N) tunneling current. The intensity of the F–N tunnel current, *J*_FN_, given by^44,45^1
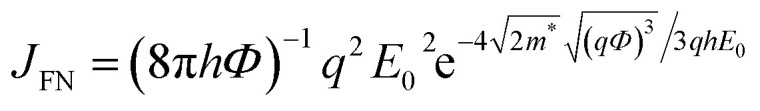
where *Φ* is the barrier height, m* is the effective mass, and *E*_0_ is the electric field applied to the blend layer. Clearly, the *J*_FN_ increases exponentially with increasing *E*_0_. The high *E*_0_ in the thinned region increases the energy of the electrons in the tunnel. The high-energy electrons in F–N tunnel collide with the electrically neutral organic molecules generating electrons–hole pairs. The electrons–hole pairs dissociate other molecules after they acquire high electric field energy. This avalanche multiplication process creates a larger leakage current. Secondly, the off-electrode Fe_3_O_4_ aggregates have a much larger surface area compared with the aggregate-free Fe_3_O_4_ NPs. Thus the former have a higher probability to catch injected electrons in the blend layer. At the same time, holes are easily injected into Fe_3_O_4_ aggregates due to the little difference between the Fermi level of Fe_3_O_4_ and the HOMO level of SY-PPV. As a result, the captured electrons and the injected holes quenched each other leading to another possible channel of leakage current.

It's well known that the holes in SY-PPV layer, having a significantly higher mobility than electrons, act as the majority carriers which decide the current density of the device. However, the probability of formation of PPs relies on electrons, the minority carriers. Since the EL is derived from S_1_ excitons, the MEL is proportional to the ^1^PP formed in device, regardless of the injected balance of carriers.^46^ Therefore, the leakage current hardly affects the reliability of using MEL to analyze the effects of spin polarization and SOC of NPs on PPs and excitons in the active layer.

### MEL curves

According to the spin polarization model, the holes are injected from anode into the magnetic nanomaterial dispersed in the active layer under a bias voltage, and then the holes are spin-polarized by magnetic nanomaterial before they hop onto host molecule.^15^ Different to the two-step injection of holes, almost all the electrons are injected from cathode directly to SY-PPV without spin polarization on Fe_3_O_4_. Ideally, the number ratio of singlet excitons to total excitons can be significantly increased from 1/4 for device without spin polarization to 1/3 for spin-polarized device. The external magnetic field can enhance the spin polarization injection on the one hand, but inhibit the ISC induced by HFI and SOC on the other hand. Either of these magnetic field effects increases the ratio of singlet excitons leading to a positive MEL. The PPV-based OLEDs generally have HFI effects which exhibit a positive MEL with characteristic fields ranging between 2–4 mT.^46–49^ If the magnetic NPs in the doped device can truly achieve spin-polarized injection of holes, the MEL of the doped device should be co-contributed by spin polarization and HFI. That is, the doped device should have a larger saturation value of MEL than the control device. In contrast, the SOC is less sensitive to an external magnetic field because it has a larger energy level splitting than HFI. So the saturated MEL of the SOC is generally less than 1%.^32^ If the SOC and HFI are dominant in the doped device, the saturation value of MEL of the doped device will be reduced compared to the control device, but the former should have a larger characteristic field than the latter.

**Fig. 5:**
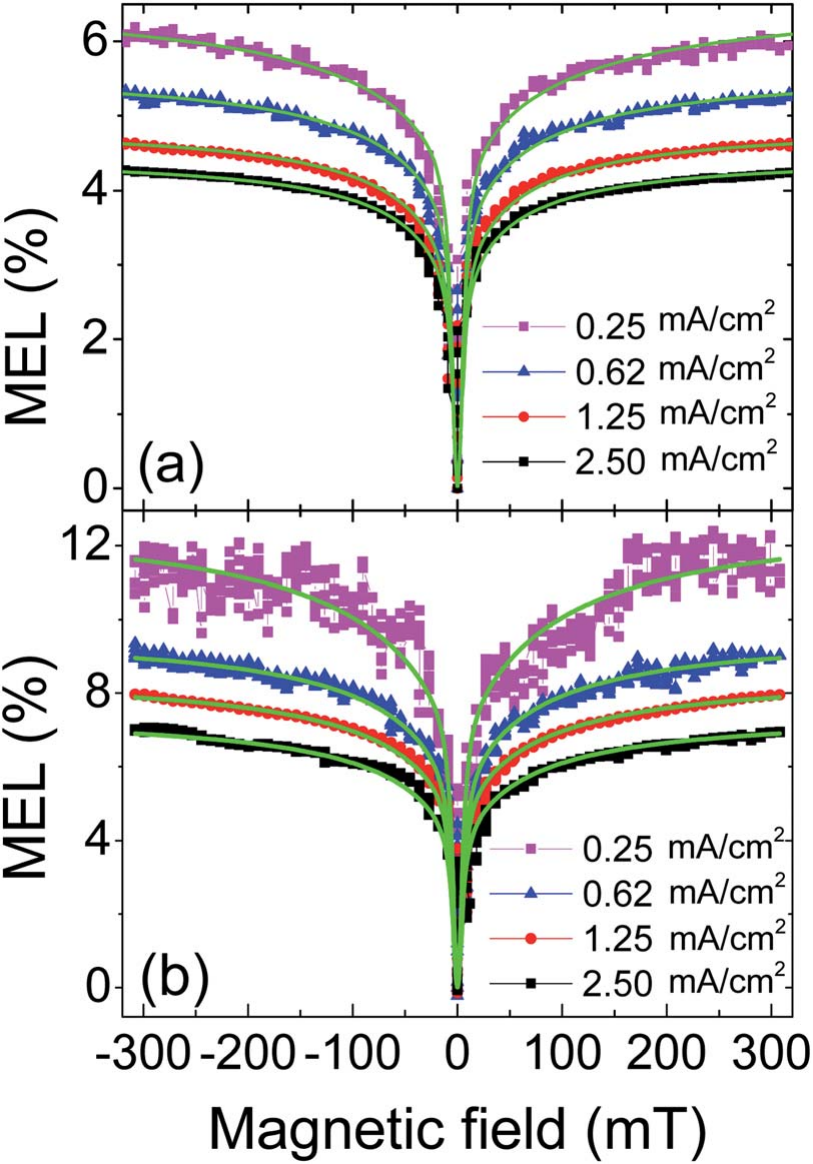
The MEL response of control device (a) and doped device (b) at different current densities at room temperature, green solid lines are fitting curves from Lorentzian empirical formula.

Based on the above inference, we measured the MEL curves at several current densities at ambient temperature both for control and doped devices, as shown in Fig. 5(a) and (b). The MEL of control device has a quite different line type to that of doped device: the former rises rapidly in low magnetic field region (0 to ∼±70 mT) and then rises slowly and becomes saturated in high field region (>∼±70 mT);^50–54^ the latter has a similar line type in low-field region to the former, but is less likely to reach saturation in high-field region. Moreover, the latter is significantly larger than the former in magnitude at 300 mT (MEL_300 mT_). This indicates that the spin related processes in doped device are obviously different to those in control device.

A modified Lorentzian empirical formula MEL = *a*_1_*B*^2^/(*B*^2^ + *B*_0_^2^) + *a*_2_*B*^2^/(|*B*| + *B*_1_)^2^ was used to fit experimental data of the two devices. The first term is the Lorentzian function,^46,50^ the characteristic field *B*_0_ is determined by the half field at half maximum (HFHM) of low-field MEL. The second term is the non-Lorentzian function,^50,55^ which is used to fit the characteristic field *B*_1_ for high-field MEL curves. The coefficients *a*_1_ and *a*_2_ represent the contributions from low-field MEL and high-field MEL, respectively. Since the spin processes of HFI, the spin polarization and the SOC can modulate the low-field MEL, it is feasible to identify them by *B*_0_. The best fitting results are the solid green lines in Fig. 5.

**Fig. 6:**
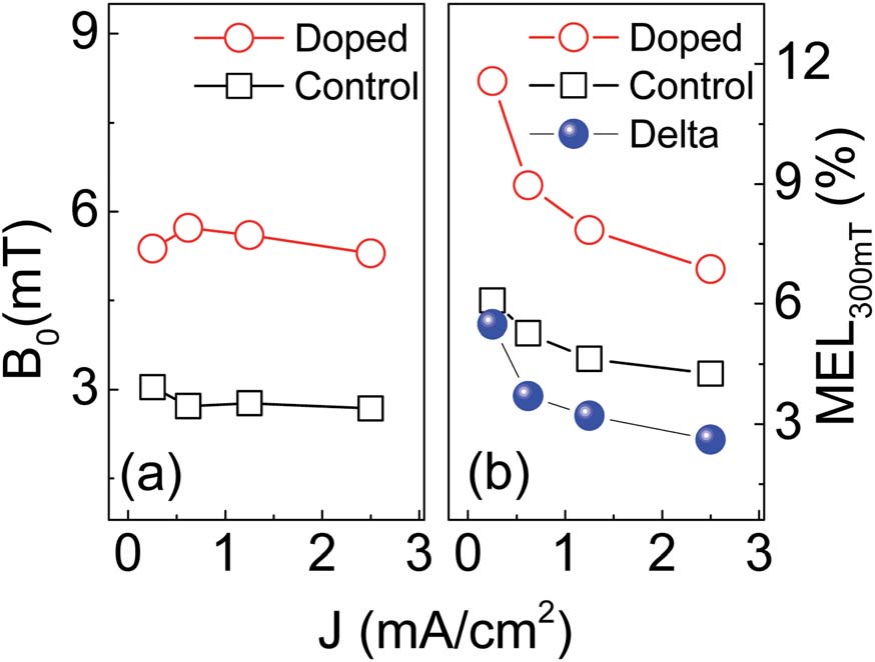
The current density dependence of characteristic magnetic field *B*_0_ (a) and the MEL_300 mT_ (b) of control and doped devices at ambient temperature.

For convenience, the *B*_0_ value and the MEL_300 mT_ at different current densities are shown in Fig. 6(a) and (b), respectively. Fig. 6(a) indicates that the *B*_0_ of the control device (∼2.7 mT) is almost unchanged as the current density increases from 0.25 to 2.50 mA cm^−2^. Generally, the *B*_0_ for HFI ranges within 2–4 mT and behaves insensitive to current density.^50^ Thus we attributed the low-field MEL of the control device to HFI. In contrast, the *B*_0_ of the doped device (∼5.5 mT) is significantly larger than that of the control device. This indicates that there is an additional process in the doped device generates positive MEL. This process should not be the SOC because the *B*_0_ of the doped device is far below the characteristic field of SOC (>30 mT).^32,33^

The MEL_300 mT_ of the doped device (red hollow circle) are significantly larger than that of the control device (black hollow square) in all current densities (Fig. 6(b)). This also does not meet the MEL characteristics of SOC. However, it can be well explained by the model of spin polarization: the MEL induced by spin-polarized and by HFI are superimposed to make the doped device have a larger MEL_300 mT_ than the control device. Since the HFI-contributed MEL saturates far below 300 mT, the difference in MEL_300 mT_ (Delta) between the doped device and control device should be the contribution of spin polarization, as shown by the blue sphere in Fig. 6(b). The Delta drops obviously as the current density increases from 0.25 to 2.50 mA cm^−2^. This can be well explained by our modified spin-polarized model. In this model, there are two channels for the injection of holes in magnetic nanoparticle doped devices: polarized-free injection channel and polarized injection channel. In the former, holes are injected directly from the anode onto the SY-PPV molecule. In the latter, holes are first injected from the anode onto the Fe_3_O_4_ NPs for spin polarization, and then injected into the SY-PPV molecule. A small current density means more percentage of holes injected into active layer *via* polarized injection channel. Oppositely, an increasing population of holes has no chance to enter the polarized injection channel to get spin polarization as the current density increases. Thus the polarized-free injection of holes becomes primary leading to a weakened MEL.

Although the existence of the SOC in the Fe_3_O_4_-doped PLEDs is ruled out, the specific reasons are still unclear. It may be caused by the following factors: (i) the atomic number of Fe is too small to generate a SOC strong enough to promote the ISC of PPs or excitons. (ii) The ISC is more sensitive to the iron-induced SOC in OSCs with donor–receptor (D–A) blend layer than in PLEDs without D–A layer. These two possible causes will be investigated in our future work.

## Experimental

The device uses poly(3,4-ethylenedioxythiophene) polystyrene sulfonate (PEDOT:PSS) as the injection & transport layer of hole, indium tin oxide (ITO) transparent conductive glass as the anode, SY-PPV as the active layer, and CsF/Al composite layer serves as the cathode with an effective light-emitting area 2 × 2 mm^2^. The device architecture is ITO/PEDOT:PSS (40 nm)/SY-PPV:Fe_3_O_4_ (80 nm, 0.5%)/CsF (2 nm)/Al (100 nm). The solution state of Fe_3_O_4_ NPs (5 nm) in toluene (10 mg mL^−1^) were purchased from NaJing Technology Corporation LTD. Additional information on these NPs were characterized in the ESI.† The TEM image of Fe_3_O_4_ NPs (Fig. S2†) suggested that the Fe_3_O_4_ NPs are well distributed at a mean size of 5 nm. The Raman and X-ray diffraction (XRD) analysis were performed at room temperature on Renishaw Invia Raman spectrometer and TD3500, respectively. The Raman shift (Fig. S3†) and XRD pattern (Fig. S4†) indicated that the NPs is almost in pure Fe_3_O_4_ phase.^4,56^ The materials of PEDOT:PSS and SY-PPV are purchased from Xi'an Bao Laite Technology Co., Ltd. Among them PEDOT:PSS and Fe_3_O_4_ NPs are pre-formed aqueous solutions and oily solutions with a same concentration of 10 mg mL^−1^. The SY-PPV solution was prepared by dissolving the fibrous SY-PPV into the organic solvent chlorobenzene. The mixture was placed on a hot plate in a dry glove box and heated and stirred for 48 hours. Then, according to the ratio of 199 : 1 in weight, the filtered SY-PPV solution and the Fe_3_O_4_ oily solution are transferred to a magnetons-free reagent bottle fixed on a copper plate. And then the reagent bottle together with copper plate was placed on a hot plate at 55 °C to perform a 24 hour heating and mixing process. After that the fully mixed SY-PPV/Fe_3_O_4_ blend solution with a concentration of 0.5 wt% was obtained. The PEDOT:PSS and SY-PPV/Fe_3_O_4_ blend layers were prepared by spin coating in ultra-clean workbench and glove box respectively. Finally, the CsF/Al cathode was evaporated in a high vacuum sample preparation system superior to 10^−5^ Pa. The film thickness was monitored by XTM/2 crystal detector made from INFICON. The control device based on pristine SY-PPV is also prepared in a same way. The TEM and EDX analysis of SY-PPV/Fe_3_O_4_ layer was performed on a JEOL JEM-2010 coupled with a JEOLEX-14053JGT energy-dispersive X-ray spectroscopy detector operated at 200 kV. The measuring instruments and methods for MEL and *J*–*B*–*V* characteristics are the same as those reported in the previous literature.^57^

## Conclusions

In summary, we used MEL fingerprint to identify whether the spin polarization and the SOC coexist in Fe_3_O_4_-doped PLEDs. Different from the reported SOC in Fe_3_O_4_-doped OSCs, there is no MEL evidence for SOC in our device. However, the MEL response confirmed the existence of spin polarization. It should be pointed out that although the spin polarization in magnetic nanoparticle-doped PLEDs contributes to electroluminescence, the current efficiency of PLEDs does not improve because the Fe_3_O_4_ NPs and their aggregates in the active layer act as a spin-polarized medium and a carrier quenching center simultaneously. This poses a serious challenge to the technical solution of using spin polarization to improve quantum efficiency of PLEDs. Evenly mixing magnetic NPs without conductivity or with ultra-low conductivity into the active layer is a possible solution to this issue.

## Conflicts of interest

There are no conflicts to declare.

## Supplementary Material

RA-009-C9RA01501A-s001
