# Supplementary material for: Using magneto-electroluminescence as a fingerprint to identify the spin polarization and spin–orbit coupling of magnetic nanoparticle doped polymer light emitting diodes
Source: RSC Adv. 2019 May 21;9(28):15845–51. doi: 10.1039/c9ra01501a (PMC9064273; doi:10.1039/c9ra01501a)
Supplement: RA-009-C9RA01501A-s001 [file RA-009-C9RA01501A-s001.pdf]

Electronic Supplementary Material (ESI) for RSC Advances.
This journal is © The Royal Society of Chemistry 2019

Supporting Information

Using Magneto-electroluminescence as a Fingerprint to Identify the Spin Polarization and Spin-orbit Coupling of Magnetic Nanoparticle Doped Polymer Light Emitting Diodes

Weiyao Jia[a,b,c], Tadaaki Ikoma[c], Lixiang Chen[a], Hongqiang Zhu[a], Xiantong Tang[a] , Fenlan Qu[a] and Zuhong Xiong*[,a,b]

Prof. Zuhong Xiong (E-mail: zhxiong@swu.edu.cn)& Dr. Weiyao Jia (E-mail: wyjia@swu.edu.cn)

a. *School of Physical Science and Technology, Southwest University, Chongqing 400715, People's Republic of China.*

b. *MOE Key Laboratory on Luminescence and Real-Time Analysis, Southwest University, Chongqing 400715, People's Republic of China.*

Prof. Tadaaki Ikoma (E-mail: ikoma@chem.sc.niigata-u.ac.jp) & Dr. Weiyao Jia (E-mail: wyjia@swu.edu.cn)

c. *Graduate School of Science and Technology, Niigata University, 2-8050 Ikarashi, Nishi-ku, Niigata 950-2181, Japan.*

Dr. Lixiang Chen, Dr. Hongqiang Zhu, MSC Xiantong Tang & MSC Fenlan Qu

a. *School of Physical Science and Technology, Southwest University, Chongqing 400715, People's Republic of China.*

Supplementaly Note:

1. Spin injection model

The spin polarization of metal magnetic nanomaterials promotes the conversion from triplet polaron pair ($^3PP_{m=0,1}$, precursor of the triplet exciton) to singlet polaron pair ($^1PP_{m=0}$, a precursor of singlet exciton) in PLEDs, which theoretically increases the R_{ST} from 1/3 to 1/2, as shown in scheme S1.

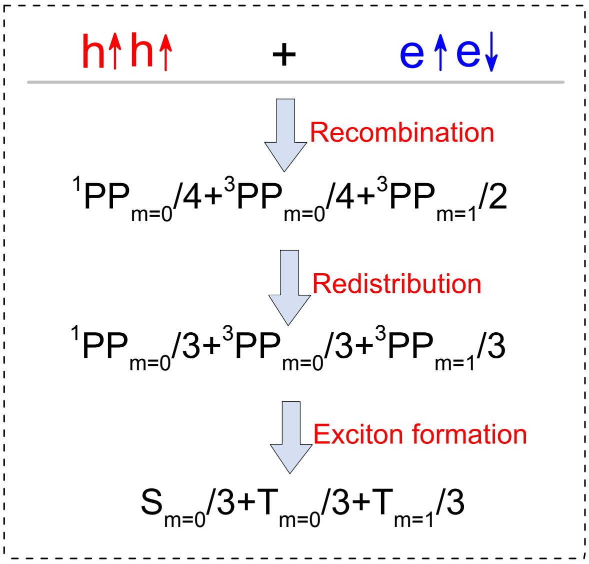

Scheme S1. Spin injection model. [1, 2]

2. EDX Spectra Analysis of blend film

The analysis of SY-PPV/ Fe_3O_4 blend film was performed using a TEM coupled with a JEOLEX-14053JGT energy-dispersive X-ray (EDX) spectroscopy detector operated at 200 kV.

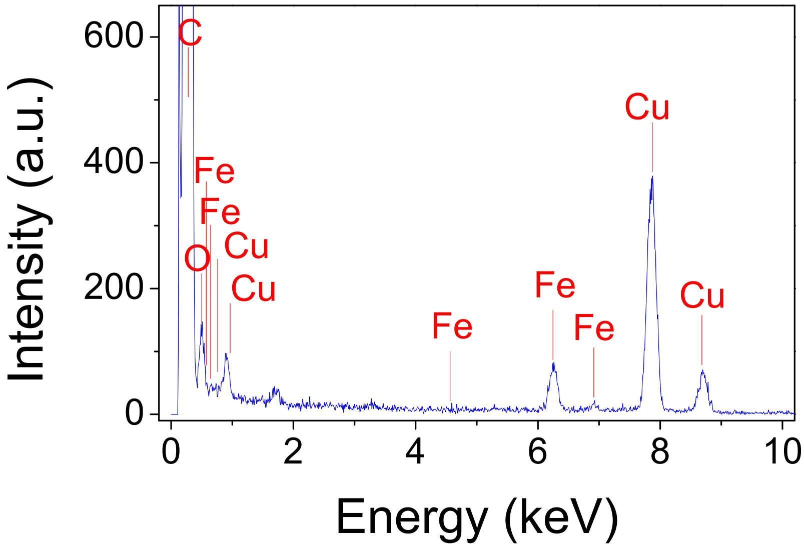

Fig.S1 EDX spectra of SY-PPV/ Fe_3O_4 blend layer. Sample was placed on a copper TEM grid for examination. Peaks corresponding to Fe and O are evident. Cu signals are from copper grid supporting blend layer.

3. Information about Fe_3O_4 nanoparticles

3.1 Transmission Electron Microscopy

The Fe_3O_4 NPs (5 nm) had been dispersed in a toluene solution at a concentration of 10 mg/mL before they were bought from NaJing Technology corporation LTD (China). Some publications involve the company's magnetic nanoparticle products. [3] A Transmission Electron Microscopy (TEM) image of Fe_3O_4 nanoparticles was provided by this company, as shown in Fig.S2.

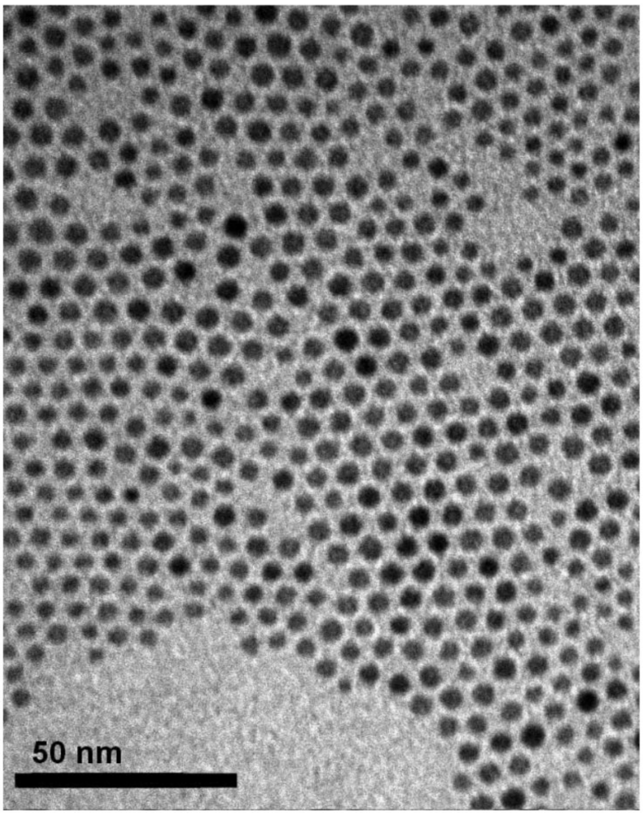

Fig.S2 TEM image of Fe_3O_4 NPs in organic solvent provided by NaJing Technology Corporation LTD.

3.3 Raman Spectra Analysis

Raman spectrum has been proved as a useful technique to differentiate various iron oxide phase, especially between Fe_3O_4 and γ-Fe_2O_3. [4] The film samples for Raman analysis were prepared by spincoating Fe_3O_4 on SiO_2 substrates. The substrate was baked in a glove box at 60 °C for 10 minutes and then used again for spin-coating, which was repeated 5 times to make the sample film thick enough. After the sample preparation, the Raman spectra were measured immediately with a single 10 s accumulation at room temperature in a Raman spectrometer (Renishaw Invia Raman spectrometer, Invia, U.K.) with the equipment of a line (λ=532 nm) from a He-Ne laser (10 mW of power on the 50 X objective). The Raman spectrum was shown in Fig. S3. Three peaks at 319, 528

and 661 cm^{-1} can be assigned to the T_{2g}, E_g and A_{1g} modes of Fe_3O_4, respectively. [5, 6] A weak peak at 470 cm^{-1} is assigned to magnon scattering of Fe_3O_4. [6] However, several additional weak peaks at 291, 413, and 614 cm^{-1} were observed. Faria et. al. reported that intense laser excitation tend to decompose the Fe_3O_4 into Fe_2O_3 during the measurement of Raman spectrum. [6] We attributed these weak peaks to E_g modes of α-Fe_2O_3 because the laser power for our sample is above 7 mW (the signal-noise ratio is very low if the power drops below 7 mW).

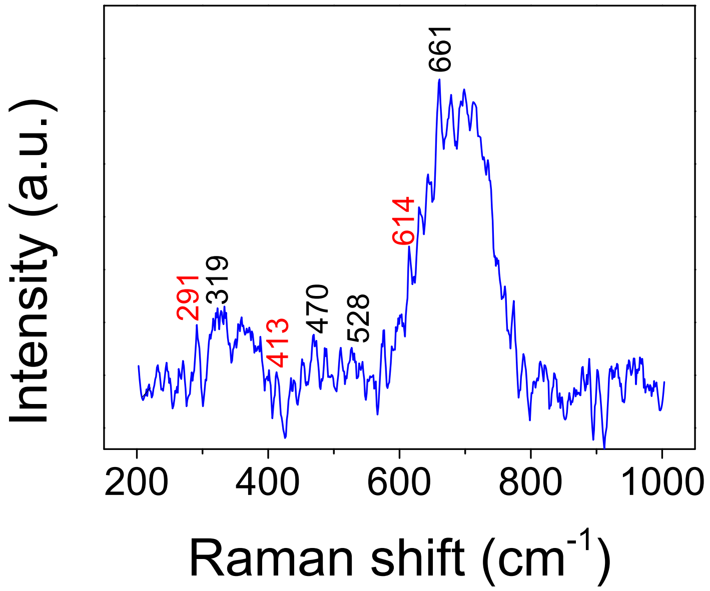

Fig.S3 Raman spectra (Renishaw Invia Raman spectrometer, Invia, U.K., resolution of 1cm^{-1}) of Fe_3O_4 nanoparticles (in solid film) on a SiO_2 substrate.

3.2 X-ray diffraction (XRD) pattern

To confirm the phase compositions of the sample of Fe_3O_4 NPs, the X-ray diffraction (XRD) analysis was performed at room temperature on TD3500 (Tongda, resolution of 0.02°) with Cu Kα radiation operated at 40 kV and 30 mA. The XRD patterns of sample were almost same to the reported diffraction peaks of polycrystalline Fe_3O_4, [1, 3] as shown in Figure 3. This further confirmed that the weak Raman peaks of α-Fe_2O_3 are originated from sample decomposition due to the use of high-power laser excitation. After the film samples for XRD analysis were prepared (similar to Raman sample), the X-ray diffraction (XRD) analysis was performed at room temperature on TD3500 (Resolution of 0.02°, Tongda, China) with Cu Kα radiation operated at 40 kV and 30 mA. The XRD patterns of sample were shown in Fig.S4.

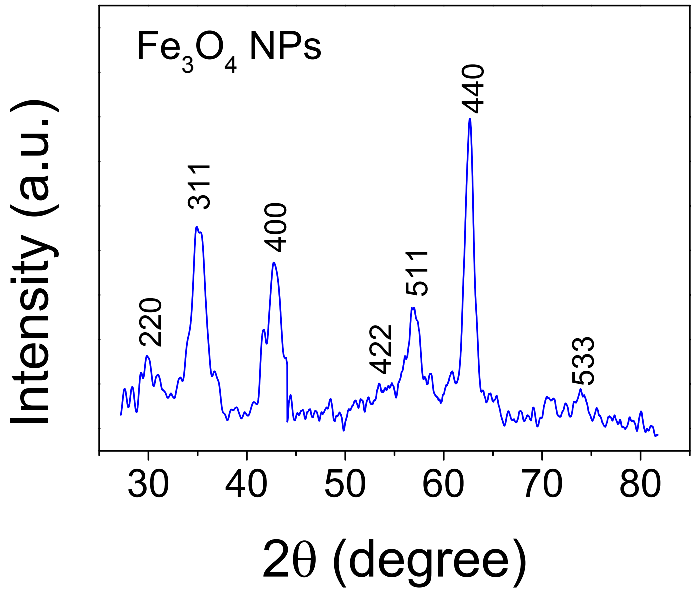

Fig.S4 XRD patterns of Fe_3O_4 NPs. (XRD analysis: TD3500, Tongda, resolution of 0.02°)

References:

1. C.-J. Sun, Y. Wu, Z. Xu, B. Hu, J. Bai, J.-P. Wang and J. Shen, *Appl. Phys. Lett.*, 2007, **90**, 232110.
2. B. Hu, Y. Wu, Z. Zhang, S. Dai and J. Shen, *Appl. Phys. Lett.*, 2006, **88**, 022114.
3. H. Kong, K. Xia, N. Ren, Y. Cui, R. Liu, Q. Li, M. Lv, J. Shi, Q. Yan, Z. Cui, C. Fan, Y. Zhu and L. Wang, *Nanoscale*, 2018, **10**, 18055-18063.
4. H. Wu, G. Wu and L. Wang, *Powder Technol.*, 2015, **269**, 443-451.
5. J.-M. Li, A. C. H. Huan, L. Wang, Y.-W. Du and D. Feng, *Phys. Rev. B*, 2000, **61**, 6876-6878.
6. D. L. A. de Faria, S. Venâncio Silva and M. T. de Oliveira, *J. Raman Spectrosc.*, 1997, **28**, 873-878.
